# Electrophysiological Characteristics of Human iPSC-Derived Cardiomyocytes for the Assessment of Drug-Induced Proarrhythmic Potential

**DOI:** 10.1371/journal.pone.0167348

**Published:** 2016-12-06

**Authors:** Wataru Yamamoto, Keiichi Asakura, Hiroyuki Ando, Tomohiko Taniguchi, Atsuko Ojima, Takaaki Uda, Tomoharu Osada, Seiji Hayashi, Chieko Kasai, Norimasa Miyamoto, Hiroyuki Tashibu, Takashi Yoshinaga, Daiju Yamazaki, Atsushi Sugiyama, Yasunari Kanda, Kohei Sawada, Yuko Sekino

**Affiliations:** 1 Japan iPS Cardiac Safety Assessment (JiCSA), Kamiyoga, Setagaya-ku, Tokyo, Japan; 2 Japanese Safety Pharmacology Society (JSPS), Showa-machi, Maebashi, Gunma, Japan; 3 Teijin Pharma Limited, Asahigaoka, Hino-shi, Tokyo, Japan; 4 Nippon Shinyaku Co., Ltd., Nishinosho-monguchi-cho, Kisshoin, Minami-ku, Kyoto, Japan; 5 Ono Pharmaceutical Co., Ltd., Yamagishi, Mikuni-cho, Sakai-shi, Fukui, Japan; 6 Eisai Co., Ltd., Tokodai, Tsukuba-shi, Ibaraki, Japan; 7 LSI Medience Corporation, Uchikanda 1-chome, Chiyoda-ku, Tokyo, Japan; 8 Astellas Pharma Inc., Miyukigaoka, Tsukuba-shi, Ibaraki, Japan; 9 Ina Research Inc., Nishiminowa, Ina-shi, Nagano, Japan; 10 Department of Pharmacology, Faculty of Medicine, Toho University, Omori-Nishi, Ota-ku, Tokyo, Japan; 11 National Institute of Health Sciences (NIHS), Kamiyoga, Setagaya-ku, Tokyo, Japan; University of Milan, ITALY

## Abstract

The aims of this study were to (1) characterize basic electrophysiological elements of human induced pluripotent stem cell-derived cardiomyocytes (hiPSC-CMs) that correspond to clinical properties such as QT-RR relationship, (2) determine the applicability of QT correction and analysis methods, and (3) determine if and how these in-vitro parameters could be used in risk assessment for adverse drug-induced effects such as Torsades de pointes (TdP). Field potential recordings were obtained from commercially available hiPSC-CMs using multi-electrode array (MEA) platform with and without ion channel antagonists in the recording solution. Under control conditions, MEA-measured interspike interval and field potential duration (FPD) ranged widely from 1049 to 1635 ms and from 334 to 527 ms, respectively and provided positive linear regression coefficients similar to native QT-RR plots obtained from human electrocardiogram (ECG) analyses in the ongoing cardiovascular-based Framingham Heart Study. Similar to minimizing the effect of heart rate on the QT interval, Fridericia’s and Bazett’s corrections reduced the influence of beat rate on hiPSC-CM FPD. In the presence of E-4031 and cisapride, inhibitors of the rapid delayed rectifier potassium current, hiPSC-CMs showed reverse use-dependent FPD prolongation. Categorical analysis, which is usually applied to clinical QT studies, was applicable to hiPSC-CMs for evaluating torsadogenic risks with FPD and/or corrected FPD. Together, this results of this study links hiPSC-CM electrophysiological endpoints to native ECG endpoints, demonstrates the appropriateness of clinical analytical practices as applied to hiPSC-CMs, and suggests that hiPSC-CMs are a reliable models for assessing the arrhythmogenic potential of drug candidates in human.

## Introduction

Numerous studies to date have used human embryonic stem cell (ESC) or induced pluripotent stem cell (iPSC)-derived cardiomyocytes (hESC/iPSC-CMs) [1–5] to both characterize the ion channels underlying the action potential (AP) and the ability of the cells to assess the arrhythmogenic potential of drugs with/without the risk of a specific form of polymorphous ventricular tachycardia termed Torsades de pointes (TdP). One platform of choice has been the multi-electrode array (MEA) technology where the extracellular field potential (FP) corresponds to the intracellular action potential (AP) as measured by the patch-clamp technique [6]. Therefore, changes in FP duration (FPD) are thought to correspond to changes in the AP duration (APD) of cardiac cells and thus to changes in electrocardiogram (ECG) parameters such as the ventricular depolarization/repolarization (QT) interval and the beat to beat (RR). However, little information is available correlating changes in MEA measured FPD and beat rate endpoints to clinical endpoints such as QT, RR, and the QT-RR relationship, or how clinical correction formulae used to minimize the impact of heart rate differences can be applied in hiPSC-CM measurements.

Heart rates vary between individuals and there is a positive correlation between the RR and QT intervals that is species specific and conventionally analyzed from QT-RR plots [7–10]. One well publicized example of the QT-RR relationship arises from the Framingham Heart study where QT interval data over varying heart rates was obtained from 5,018 participants, ranging from 28 to 62 years of age [9]. Similarly, beat rate and FPD in hiPSC-CMs show variation from preparation to preparation, and changes after application of test compounds. However, the relation between FPD and interspike interval (ISI) in hiPSC-CMs, and the correlation of this relationship with that of the QT-RR relation found in humans has not been reported previously.

Drug-induced prolongation of the QT interval in the ECG recording is widely accepted as a surrogate marker of arrhythmogenicity in clinical trials. A primary determinant of drug-induced QT prolongation is inhibition of the rapid delayed rectifier current (*I*_Kr_) mediated by the human-ether-à-go-go related gene channels. It is well known that *I*_Kr_ inhibitors such as E-4031 and dofetilide show reverse use-dependency; e.g. repolarization is preferentially prolonged at slow heart rates in human [11–14]. Thus, it is important to offset the influence of heart rate on the QT interval as has been proposed and widely used by Fridericia (QTcF) [8] or Bazett (QTcB) [7]. However, there is no evidence that either methodologies (or others) are applicable to correcting of FPD from hiPSC-CMs of varying beat rates. Further, although FPD prolongation with *I*_Kr_ inhibitors is well characterized in hiPSC-CMs [2, 4], reports addressing reverse use-dependent effects in these cells do not currently exist.

The ICH E14 document provides guidance on clinically evaluating QT/QTc prolongation and proarrhythmic potential of test compounds in human subjects [15]. This guideline recommends categorical analyses of QT/QTc interval data based on the number and percentage of patients meeting or exceeding several predefined criteria in thorough QT (TQT) studies. An absolute QTc interval of a > 500 ms, and change from baseline in QTc interval of > 30 or 60 ms are conventionally used as the criteria for evaluating the arrhythmogenicity of test compounds [15]. A number of MEA-based hiPSC-CM studies have reported that drugs with TdP risks prolonged FPD/FPDc and induced arrhythmogenic waveforms such as early afterdepolarizations (EADs) and triggered activity (TA) [1–4]. While a categorical analysis has been performed to evaluate the repolarization delay in hiPSC-CMs [4], the relationship between FPD prolongation and arrhythmogenicity in hiPSC-CMs has yet to be defined by this methodology.

The Japan iPS Cardiac Safety Assessment (JiCSA) initiative has conducted a series of MEA-based experiments using hiPSC-CMs with the National Institute of Health Sciences (NIHS), the Japanese Safety Pharmacology Society (JSPS), the Consortium for Safety Assessment using Human iPS Cells (CSAHi), and the Comprehensive in vitro Proarrhythmia Assay (CiPA) program to develop a standardized protocol [16][17]. In the present study, experiments were carried out at four independent facilities, and data obtained using a common protocol was analyzed, focusing on the relationship between FPD and beating rate. Categorical analyses used for the clinical TQT study were conducted to explore potential indices of TdP risks with hiPSC-CM using the MEA assay. Here, we demonstrate for the first time that hiPSC-CMs likely share similar electrophysiological properties with human hearts, suggesting that hiPSC-CMs may hold promise for predicting arrhythmogenic potential. This would represent an important advancement for drug development.

## Materials and Methods

Datasets for this study were obtained from Eisai Co., Ltd., Nippon Shinyaku Co., Ltd., Ono Pharmaceutical Co., Ltd., and Teijin Pharma Limited, abbreviated below as A, B, C and D, respectively. The datasets were obtained using the common protocol reported previously [18].

### Cell culture and plating

Human iPSC-derived cardiomyocytes (iCells Cardiomyocytes, lot# 1093227) were purchased from Cellular Dynamics International (Madison, WI, USA). The cells were thawed and suspended in iCell Cardiomyocytes Plating Medium (Cellular Dynamics International), then plated (approximately 2 × 10^6^ cells/well) in 6-well tissue-culture plates coated with 0.1% gelatin. After two days of incubation at 37°C under an atmosphere of 5% CO_2_, the plating medium was replaced with iCell Cardiomyocytes Maintenance Medium (Cellular Dynamics International); this medium was used as the culture medium throughout the experiments and was replaced every 1 to 3 days. The cells were cultured for 7 to 14 days in a 5% CO_2_ incubator at 37°C and then re-plated onto MEA probes (MED-P515A or MED-PG515A; Alpha Med Scientific, Osaka, Japan).

The recording areas of the MEA probes were coated with fibronectin (Becton Dickinson, Franklin Lakes, NJ, USA; Invitrogen, Carlsbad, CA, USA; Corning, Corning, NY, USA). Fibronectin was dissolved in distilled water or Dulbecco's phosphate-buffered saline (PBS) [−] to give 50 μg/mL, the recording areas were covered with 2 μL fibronectin solution, and then the MEA probes were incubated at 37°C for at least 1 h. The cells cultured in the 6-well tissue-culture plates were dispersed with 0.25% trypsin–EDTA (Wako, Osaka, Japan) or TrypLE Select (Invitrogen), and then resuspended in the maintenance medium. After discarding the fibronectin solution from the MEA probes without complete drying, the cell suspension at a density of 3.0 × 10^4^ cells in a 2 μL droplet was re-plated onto the fibronectin-coated area of each MEA probe and incubated for 1 to 3 h in a 5% CO_2_ incubator at 37°C. Each well of the MEA probes was then filled with maintenance medium at a volume of 1 mL/well. The medium was replaced every 1 to 3 days thereafter, and the cells were cultured for 5 to 14 days to obtain high density cardiomyocyte-sheets with spontaneous and synchronous electrical activities.

### Recording of field potentials (FPs)

**Fig 1:**
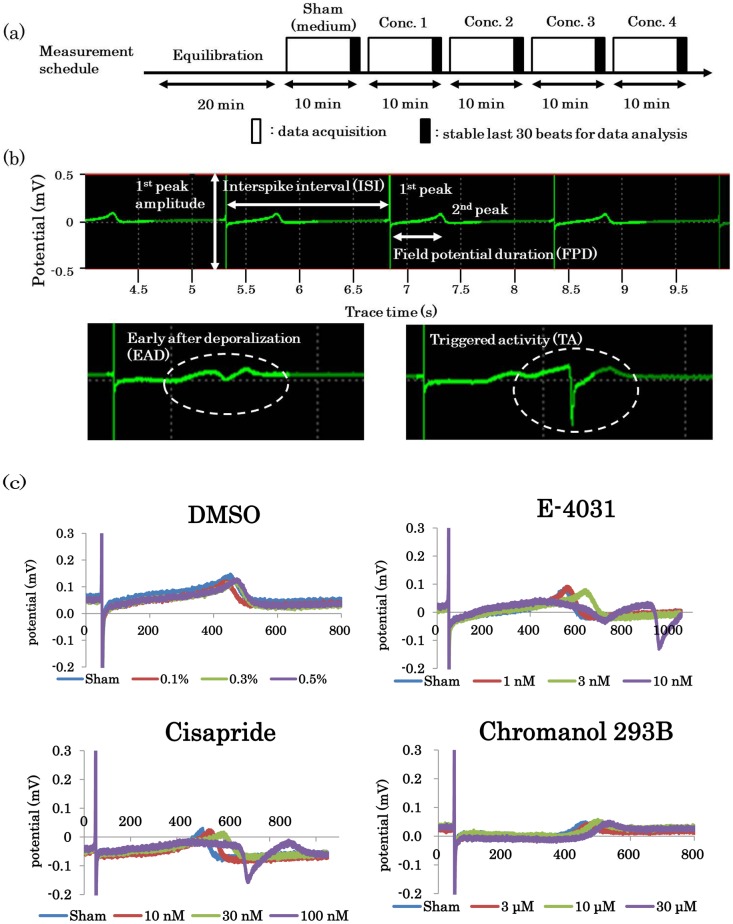
Assay design and representative field potential (FP) waveforms before/after drug application. Schematic depicting the measurement schedule for the hiPSC-CM/MEA assay (Fig 1a). The upper panels show FP waveforms and parameters, and the lower panels indicate the arrhythmogenic waveform of EAD (left, dashed circle) and TA (right, dashed circle) (Fig 1b). Example FP waveforms after application of DMSO, E-4031, cisapride or chromanol 293B (Fig 1c).

Prior to recording FPs, the cardiomyocyte-sheets were equilibrated for at least 30 min in a 5% CO_2_ incubator at 37°C in fresh culture medium. The MEA probes were then transferred to a FP measurement apparatus (MED64 system; Alpha Med Scientific, Osaka, Japan) and covered with a lid through which humidified gas containing 5% CO_2_ was provided. Using a calibrated microprobe thermometer (BAT-12, Physitemp Instruments Inc., Clifton, NJ, USA), the actual temperature of the medium 1–2 mm above the electrodes of the MEA probes was adjusted to provide readings of 36.5 to 37.0°C at each facility. Analogue FP signals from the spontaneously beating cardiomyocyte-sheets were acquired through a 0.1-Hz high-pass filter and a 5-kHz low-pass filter, and digitized at 20 kHz using the MED64 system [18]. The stability and constancy of the waveforms, ISI and FPD were confirmed for at least 20 min before a 10 min recording with sham treatment (addition of medium only) (see “sham” in Fig 1a). Cardiomyocyte-sheets were not used for further analyses if FPDcF (FPD corrected with Fridericia's formula: FPDcF = FPD/(ISI)^0.33^) and beating rates (beats per minute) did not meet the criteria described below. Compound stock solutions were prepared using DMSO at 1000-fold the target concentrations of the test compounds. The stock solutions were diluted with the maintenance medium to obtain the appropriate compound concentrations, then 1/10^th^ to 1/50^th^ the volume of the medium in the wells was exchanged with an equal volume of the compound-containing medium. The compounds were applied in a cumulative manner to the wells. The concentration of DMSO was 0.5–0.6% at the highest concentration of the test compounds. At each concentration, the FP waveforms were recorded for 10 min, and measurement was extended for up to 20 min if the readouts did not stabilize by 10 min. FPD (ms) and ISI (ms) were analyzed using Mobius QT software designed by WitWerx (Santa Cruz, CA, USA) (Fig 1b). When EAD/TAs were observed (Fig 1c), the FP recording was stopped and the compound at a higher concentration was not applied.

### FP data analysis

Baseline FP waveforms that met the following criteria were used for analysis. Briefly, waveforms in which the first positive or negative peak amplitude was ≥ ± 200 μV, the second peak amplitude was ≥ 20 μV, FPDcF was 340–450 ms, and the interspike interval (ISI) was < 1715 ms, corresponding to above 35 bpm (beats per minute), were used for further analysis (Fig 1b). The values of ISI and FPD were averaged, using either the last 30 beats or 30 beats at time points which showed stable ISI and FPD. In the FP signals, EAD and TA were defined as slow negative spikes and a sharp deflection with an amplitude ≥ ± 100 μV, respectively (Fig 1c). EAD, TA or both usually appear during or after the second positive deflection [18]. ISI, FPD and FPDcF were not calculated for concentrations in which EAD or TA were observed.

**Fig 2:**
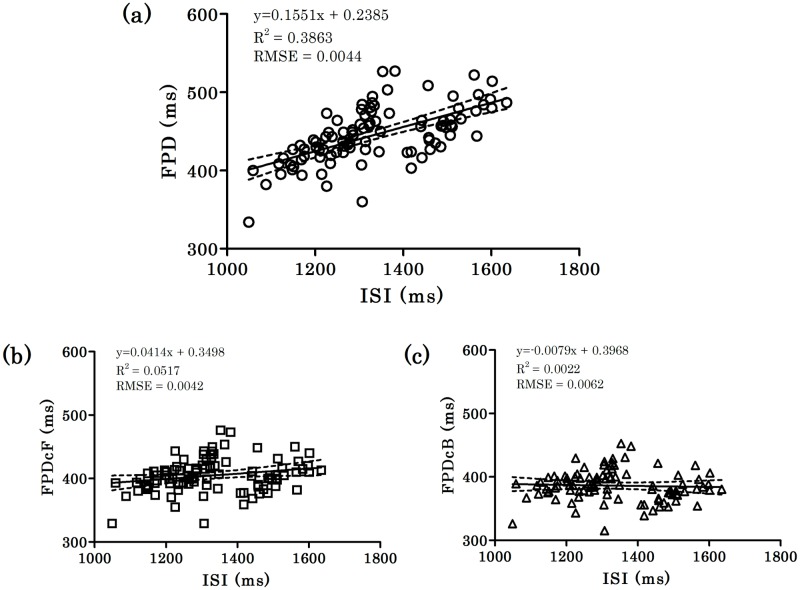
Relationship between field potential duration (FPD)/corrected FPD (FPDc) and interspike interval (ISI) of 96 samples of hiPSC-CM. The data in sham treatment from 4 facilities were plotted (n = 96) in Fig 2a (FPD-ISI), Fig 2b (FPDcF-ISI) and Fig 2c (FPDcB-ISI), respectively. The solid and dashed lines indicate the linear regression line and 95% confidence bands, respectively. The equation, R^2^ value, and root mean squared prediction error (RMSE) are shown in the figures.

**Fig 3:**
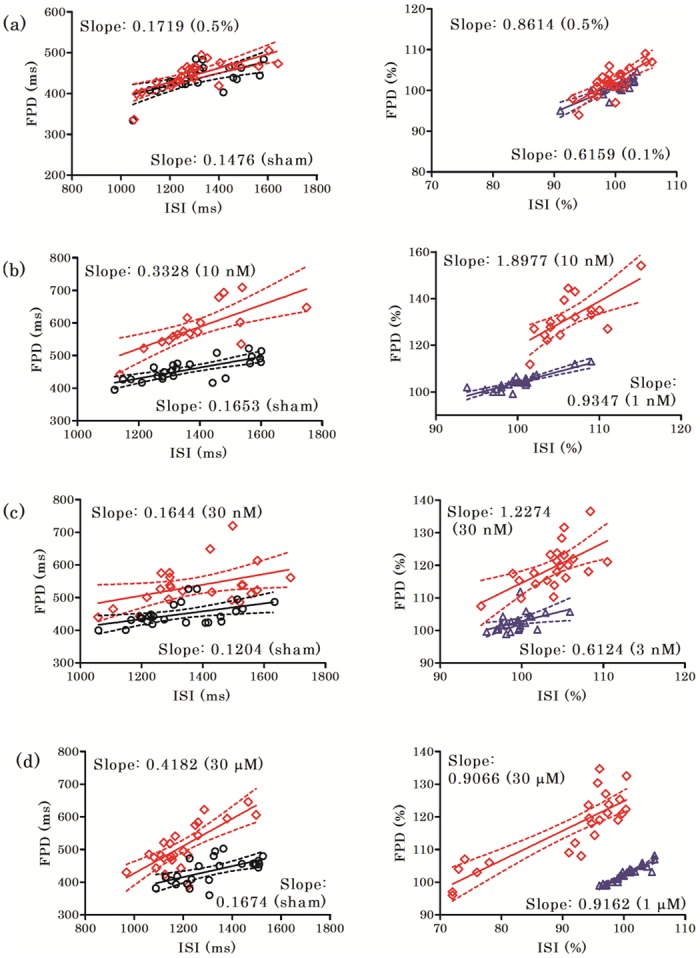
Effects of *I*_Kr_ and *I*_Ks_ inhibitors on field potential duration (FPD)-interspike interval (ISI) plots. FPD-ISI plots are shown before and after compound application both in terms of absolute values (left) and % change (right), for DMSO (Fig 3a), E-4031 (Fig 3b), cisapride (Fig 3c) and chromanol 293B (Fig 3d). The solid lines and dashed lines indicate the linear regression lines and 95% confidence bands, respectively. Data for the middle concentrations are omitted for clarity. ‘Slope’ refers to the slope of the regression line.

**Fig 4:**
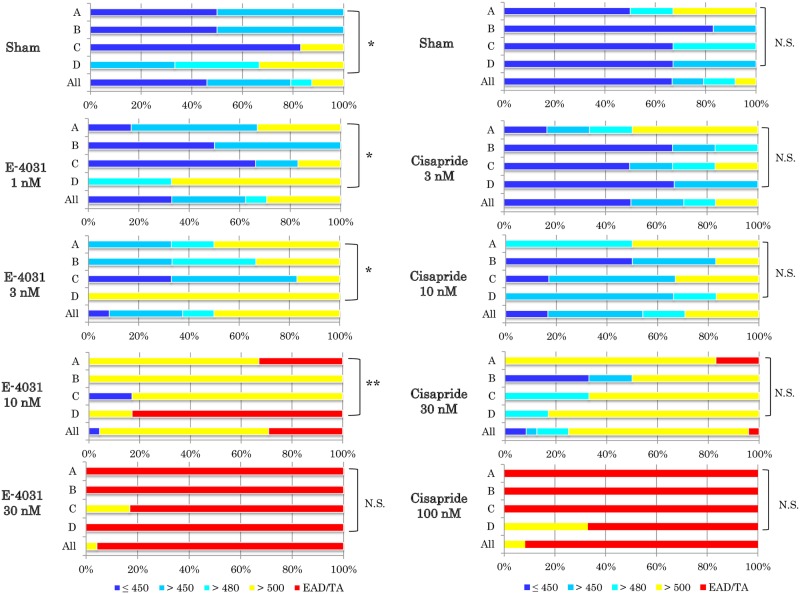
Categorical analyses of absolute FPD values after E-4031 and cisapride application. The absolute FPD values after E-4031 and cisapride application (24 data sets, 6 data per facility) were categorized in reference to ICH-E14 guideline; absolute QT interval ≤450, >450, >480, >500 ms and EAD/TAs, respectively. *P<0.05, **P<0.01 Kruskal-Wallis test. EAD; early afterdepolarization, TA; triggered activity.

As previously mentioned, total 96 data equally from 4 facilities each were used for the analysis (24 data per facility). To investigate the relationship between FPD and ISI (Fig 2), and the use-dependent effects of test compounds on FPD prolongation (Fig 3), linear regressions with a 95% confidence bands were calculated on a FPD-ISI plot using GraphPad Prism version 5.00 for Windows (GraphPad Software, San Diego, CA, USA). The root mean squared prediction error (RMSE) was used to show the level of accuracy of the equation predicted for the observed data (Fig 2). With categorical data analysis of FPD, inter-facilities differences were investigated with Kruskal-Wallis test (Fig 4).

### Drugs

E-4031 was obtained from Wako Pure Chemical Industries. Chromanol 293B and cisapride was purchased from Sigma-Aldrich (St. Louis, MO, USA). These drugs were dissolved in dimethyl sulfoxide (DMSO) (Wako, Japan).

## Results

### Effects of *I*_Kr_ and *I*_Ks_ inhibitors on iPSC-CMs using the MEA assay

Fig 1a and 1b shows the experimental design of the MEA assay and representative FP waveforms after drug application. Fig 1c shows superimposed FP waveforms after application of DMSO and each drug. The effects of vehicle on FP were examined by applying DMSO in a cumulative manner to a final concentration of 0.5–0.6%. FPD was slightly altered by the addition of DMSO, but the ratios (%) of FPD prolongation were recorded less than 5% in each facility at the highest concentration of DMSO. E-4031 and cisapride are both *I*_Kr_ inhibitors and prolonged FPD in a concentration-dependent manner. EADs and/or TAs were observed at concentrations ranging from 10 nM to 100 nM for E-4031, and at concentrations from 30 to 300 nM for cisapride. Chromanol 293B, a *I*_Ks_ inhibitor, also prolonged FPD in a concentration-dependent manner, but EADs and/or TAs were not observed at concentrations up to 30 μM. The detailed data are presented in the supplements (S1 and S2 Tables).

### FPD-ISI relationship

To explore the clinically-relevant electrophysiological characteristics of hiPSC-CMs, the relationship between FPD and ISI was analyzed using data from 96 samples obtained from 4 facilities (Fig 2a). Longer values for ISI correlated with a longer FPD. Linear regression analysis of the FPD-ISI plot provided the formula: y = 0.1551x + 0.2385 (R^2^ = 0.3863 and RMSE = 0.0044). The obtained regression coefficient is close to that obtained using the human QT-RR regression model in the Framingham Heart Study (QT_C_ = QT + 0.154[1 − RR]) [9].

We further examined whether both Fridericia’s and Bazett’s corrections are applicable for correcting FPD for ISI in hiPSC-CMs. Both formulae could adequately correct FPD for ISI (Fridericia’s: Slope factor = 0.0414; Bazett’s: Slope factor = -0.0079) compared with the absolute FPD (Slope factor = 0.1551) (Fig 2b and 2c). Since Bazett’s formula was generally likely to over-correct FPD at slow ISI, and it may under-estimate FPD prolongation, Fridericia’s formula was used in the categorical data analysis described in S5 Table.

### Effects of *I*_Kr_ and *I*_Ks_ inhibitors on the FPD-ISI plots

Use-dependent effects of FPD prolongation were investigated by analyzing the FPD-ISI plots of DMSO, E-4031, cisapride and chromanol 293B (Fig 3 and S3 Table). The regression coefficient values were similar before and after DMSO treatment both in terms of the absolute (ms) values and the ratios of the absolute values (% change) (Slope factors from 0.1476 (Sham) to 0.1719 (0.5%) for absolute FPD, and from 0.6159 (0.1%) to 0.8614 (0.5%) for % change).

The application of either E-4031 or cisapride to hiPSC-CM preparations increased the regression coefficients of the FPD-ISI plots in a concentration-dependent manner for both the absolute value and the % change (E-4031: Slope factors from 0.1653 (Sham) to 0.3328 (10 nM) for absolute FPD, from 0.9347 (1 nM) to 1.8977 (10 nM) for % change; Cisapride: Slope factors from 0.1204 (Sham) to 0.1644 (30 nM) for absolute FPD, from 0.6124 (3 nM) to 1.2274 (30 nM) for % change).

Chromanol 293B increased the regression coefficients in a concentration-dependent manner when the absolute values were analyzed (Slope factors: from 0.1674 (Sham) to 0.4182 (30 μM)), whereas concentration-dependent changes were not apparent in the slope of the regression line fit to the data when analyzed by % change (Slope factors: from 0.9162 (1 μM) to 0.9066 (30 μM)). These results indicated that E-4031 and cisapride, but not chromanol 293B, induced FPD prolongation in a reverse use-dependent manner.

### Categorical data analysis of FPD, delta FPD and delta FPDcF

Categorical analysis is conventionally used in clinical TQT studies [15]. To assess the effects of *I*_Kr_ inhibitors on FPD prolongation and the incidence of EAD/TAs, 24 data sets (6 data per facility) were categorized in reference to ICH-E14 guideline; absolute QT interval ≤450, >450, >480, >500 ms and EAT/TAs, respectively (Tables 1 and 2 and Fig 4).

**Table 1:** Categorical analyses of absolute FPD values after E-4031 application.

Facility	FPD Category (ms)	Sham	E-4031 (nM)				
			1	3	10	30	100
A	≤ 450	50%(3/6)	17%(1/6)	0%(0/6)	0%(0/6)	N.D.	
	> 450	50%(3/6)	50%(3/6)	33%(2/6)	0%(0/6)		
	> 480	0%(0/6)	0%(0/6)	17%(1/6)	0%(0/6)		
	> 500	0%(0/6)	33%(2/6)	50%(3/6)	67%(4/6)		
	EAD/TA	-	-	-	33%(2/6)	100%(6/6)	
B	≤ 450	50%(3/6)	50%(3/6)	0%(0/6)	0%(0/6)	N.D.	
	> 450	50%(3/6)	50%(3/6)	33%(2/6)	0%(0/6)		
	> 480	0%(0/6)	0%(0/6)	33%(2/6)	0%(0/6)		
	> 500	0%(0/6)	0%(0/6)	33%(2/6)	100%(6/6)		
	EAD/TA	-	-	-	-	100%(6/6)	
C	≤ 450	83%(5/6)	67%(4/6)	33%(2/6)	17%(1/6)	0%(0/6)	N.D.
	> 450	0%(0/6)	17%(1/6)	50%(3/6)	0%(0/6)	0%(0/6)	
	> 480	0%(0/6)	0%(0/6)	0%(0/6)	0%(0/6)	0%(0/6)	
	> 500	17%(1/6)	17%(1/6)	17%(1/6)	83%(5/6)	17%(1/6)	
	EAD/TA	-	-	-	-	83%(5/6)	100%(6/6)
D	≤ 450	0%(0/6)	0%(0/6)	0%(0/6)	0%(0/6)	N.D.	
	> 450	33%(2/6)	0%(0/6)	0%(0/6)	0%(0/6)		
	> 480	33%(2/6)	33%(2/6)	0%(0/6)	0%(0/6)		
	> 500	33%(2/6)	67%(4/6)	100%(6/6)	17%(1/6)		
	EAD/TA	-	-	-	83%(5/6)	100%(6/6)	
All	≤ 450	46%(11/24)	33%(8/24)	8%(2/24)	4%(1/24)	N.D.	
	> 450	33%(8/24)	29%(7/24)	29%(7/24)	0%(0/24)		
	> 480	8%(2/24)	8%(2/24)	13%(3/24)	0%(0/24)		
	> 500	13%(3/24)	29%(7/24)	50%(12/24)	67%(16/24)		
	EAD/TA	-	-	-	29%(7/24)	96%(23/24)	100%(24/24)

N.D., Not determined because of the incidence of EAD/TAs. The parenthesis represents number of positive cells compared to the total number of cells. Yellow-filled boxes represent the incidence of EAD/TAs, gray-filled boxes indicate 'not tested'. Sham indicates treatment with medium. The data were rounded off to the whole number.

**Table 2:** Categorical analyses of absolute FPD values after cisapride application.

Facility	FPD Category (ms)	Sham	Cisapride (nM)				
			3	10	30	100	300
A	≤ 450	50% (3/6)	17%(1/6)	0% (0/6)	0% (0/6)	N.D.	
	> 450	0% (0/6)	17%(1/6)	0% (0/6)	0% (0/6)		
	> 480	17%(1/6)	17%(1/6)	50% (3/6)	0% (0/6)		
	> 500	33%(2/6)	50% (3/6)	50% (3/6)	83%(5/6)		
	EAD/TA	-	-	-	17%(1/6)	100%(6/6)	
B	≤ 450	83%(5/6)	67%(4/6)	50% (3/6)	33%(2/6)	N.D.	
	> 450	17%(1/6)	17%(1/6)	33%(2/6)	17%(1/6)		
	> 480	0% (0/6)	17%(1/6)	0% (0/6)	0% (0/6)		
	> 500	0% (0/6)	0% (0/6)	17%(1/6)	50% (3/6)		
	EAD/TA	-	-	-	-	100%(6/6)	
C	≤ 450	67%(4/6)	50% (3/6)	17%(1/6)	0% (0/6)	N.D.	
	> 450	0% (0/6)	17%(1/6)	50% (3/6)	0% (0/6)		
	> 480	33%(2/6)	17%(1/6)	0% (0/6)	33%(2/6)		
	> 500	0% (0/6)	17%(1/6)	33%(2/6)	67%(4/6)		
	EAD/TA	-	-	-	-	100%(6/6)	
D	≤ 450	67%(4/6)	67%(4/6)	0% (0/6)	0% (0/6)	0% (0/6)	N.D.
	> 450	33%(2/6)	33%(2/6)	67%(4/6)	0% (0/6)	0% (0/6)	
	> 480	0% (0/6)	0% (0/6)	17%(1/6)	17%(1/6)	0% (0/6)	
	> 500	0% (0/6)	0% (0/6)	17%(1/6)	83%(5/6)	33%(2/6)	
	EAD/TA	-	-	-	-	67%(4/6)	100%(6/6)
All	≤ 450	67%(16/24)	50%(12/24)	17% (4/24)	8% (2/24)	0% (0/24)	
	> 450	13%(3/24)	21%(5/24)	38%(9/24)	4% (1/24)	0% (0/24)	
	> 480	13%(3/24)	13%(3/24)	17%(4/24)	13%(3/24)	0% (0/24)	
	> 500	8%(2/24)	17%(4/24)	29%(7/24)	71%(17/24)	8%(2/24)	
	EAD/TA	-	-	-	4%(1/24)	92%(22/24)	100%(24/24)

N.D.; Not determined because of the incidence of EAD/TAs. The parenthesis represents number of positive cells compared to the total number of cells. Yellow-filled boxes represent the incidence of EAD/TAs, gray-filled boxes indicate 'not tested'. Sham indicates treatment with medium. The data were rounded off to the whole number.

With E-4031, the categorical distributions were different among facilities in sham treatment, however most preparations (79%) were categorized in the group below 480 ms (19/24), but 3 preparations showed FPD longer than 500 ms. The application of 3 nM E-4031 resulted in 50% of the preparations (12/24) from all 4 facilities being categorized in the group >500 ms for absolute FPDs, but no EAD/TAs were observed (also see Fig 4 and Table 1). At 10 nM E-4031, 67% of the preparations (16/24) were categorized in the >500 ms group without EAD/TAs, whereas 7 preparations showed EAD/TAs. At 30 nM E-4031, 96% of the preparations (23/24) developed EAD/TAs (see Fig 4 and Table 1).

With cisapride, there were not significant differences in categorical distributions among facilities in sham treatment. Most preparations (79%) were categorized in the group below 480 ms (19/24), although 2 preparations showed FPD longer than 500 ms. When 10 nM cisapride was applied, 29% of the preparations (7/24) from all 4 facilities were categorized in the >500 ms group for absolute FPDs, but no EAD/TAs were observed (see Fig 4 and Table 2). At 30 nM cisapride, 71% of the preparations (17/24) were categorized in the >500 ms group without EAD/TAs, and 1 preparation showed EAD/TAs. At 100 nM cisapride, 92% of the preparations (22/24) developed EAD/TAs (see Fig 4 and Table 2). Consequently, it was found that most FPDs from all faculties were categorized in the >500 ms group at concentrations just before EAD/TAs occurrence.

Categorical data analysis of delta FPD and delta FPDcF in the presence of E-4031 or cisapride are shown in S4 and S5 Tables, respectively. The analyses showed that both delta FPD and delta FPDcF increased in a concentration-dependent manner, and most delta FPD and delta FPDcF results from all faculties were categorized in the >60 ms group at concentrations just before EAD/TAs occurrence.

## Discussion

The major findings regarding the characteristic hiPSC-CM electrophysiology are as follows. 1) The regression coefficient obtained from FPD-ISI plots of hiPSC-CMs was similar to those provided by the QT-RR regression model obtained from the Framingham Heart Study [9]. 2) The influence of the beating rate on the FPD of hiPSC-CMs is reduced by applying correction formulae appropriate for human ECGs. 3) A reverse use-dependent prolongation of FPD by *I*_Kr_ inhibitors was shown in hiPSC-CMs by analyzing FPD-ISI plots, in accordance with the results obtained from a human study [13]. 4) A categorical analysis used for a clinical TQT study was also applicable to hiPSC-CMs for evaluating arrhythmogenic risks with FPD and/or FPDc.

MEA-based hiPSC-CM FP measurements were conducted so as to minimize variability among facilities; a common protocol was used across 4 facilities with a single production lot of hiPSC-CMs and consistent media composition. Moreover, the medium temperature for the assays was strictly standardized among facilities. The present data were similar to that of a previous study [4] and showed similar % changes of ISI values among the facilities but variable absolute values (detailed data obtained in the current study are described in S1 and S2 Tables). The source of the absolute variation is unclear but might arise from inevitable inter-preparation differences that may thus affect the mechanisms for controlling responses; either the beating rate, within local pace-making cells or slight differences in the ventricular-type cells, which are the major cell-type contained in the cell sheets prepared for the FP recording. Regardless of the source of variation, the primary point is that the differences could be accounted for through normalization. This would be similar to the relationship between pace-making in sinus nodes and the QT interval in ventricular cells in human heart.

The relationship between FPD and ISI, which corresponds to the QT-RR relationship in the human ECG, was also investigated. The regression coefficient (FPD = 0.1551 x ISI + 0.2385) of hiPSC-CMs was interestingly close to those of QT-RR regression model obtained from Framingham Heart Study ECGs. The hiPSC-CM coefficient in this study was obtained from 96 cardiomyocyte preparations prepared from a single production lot differentiated from a single donor. Individual cellular variation was minimized as each preparation was composed of 30,000 hiPSC-CMs, and preparation-preparation variation was minimized by analyzing the FPD-ISI relationship of all 96 preparations. As this data was from a single donor, it would of interest to analyze the regression coefficients in a similar manner across multiple donors to determine the degree to which hiPSC-CM FPD-ISI matched donor ECG QT-RR or whether hiPSC-CM FPD-ISI could be an emergent evaluation criteria for the quality control of hiPSC-CMs in assessing drug-induced proarrhythmic potential in human.

The present study also showed that both Fridericia’s [QTcF = QT/(RR)^0.33^] and Bazett’s [QTcB = QT/(RR)^0.5^] formulae, which are generally used for interpreting clinical ECGs, were fairly effective in correcting FPD for ISI in the FP recordings of hiPSC-CMs. However, it is likely that Fridericia’s formula under-corrects and that Bazett’s formula over-corrects FPD at slow ISI under the present experimental conditions. We found that the formula QTc = QT/(RR)^0.47^ accurately corrected FPD for ISI in this study. This QT correction formula interestingly has a similar power function to that of QTc = QT/(RR)^0.45^ obtained by analyzing data from an infant population [19]. Thus, the present study suggests that QT corrections used in analyzing human ECGs are likely applicable to FPD corrections of hiPSC-CMs. Furthermore, that a more appropriate correction formula may be required for each FP recording experiment in order to more accurately assess drug-induced proarrhythmia risk. Alternatively, an experimental protocol to pace the hiPSC-CMs with electrical stimulations could also be developed.

*I*_Kr_ inhibitors such as E-4031 and cisapride prolong repolarization preferentially at slow heart rates in rabbit and canine Purkinje fiber models [14, 20], and in human ventricular preparations [11]. Consistent with these reports, both E-4031 and cisapride prolonged FPD in a reverse use-dependent manner, indicating that iPS-CMs are a good alternative model for animal and human heart for assessing *I*_Kr_ inhibitors. In contrast to the *I*_Kr_ inhibitors, chromanol 293B, a *I*_Ks_ inhibitor, prolonged FPD in a rate-independent manner. The rate-dependent effects of chromanol 293B are presently unclear. In human and guinea pig ventricular myocytes, the drug was found to prolong APD_90_ by about 30%, independent of the underlying pacing rate [21]. On the other hand, Bauer et al. reported that chromanol 293B prolonged effective refractory periods in a rate-dependent manner in dogs with subacute myocardial infarction [22]. Although the reasons for the different outcomes regarding the reverse-rate dependent effects of chromanol 293B are not clear at the moment, these controversial results may result from variable responsiveness between species, or from variable methodologies used in these reports.

Since the hiPSC-CMs showed a human-relevant QT-RR relationship, we analyzed the FPD prolongation and incidence of EAD/TAs in hiPSC-CMs according to the categorical methods recommended in the ICH-E14 guideline [15]. Both *I*_Kr_ inhibitors prolonged FPD/FPDcF in a concentration-dependent manner. In addition, the absolute FPD (500 ms), delta FPD and/or delta FPDcF (60 ms, see S4 and S5 Tables) act as thresholds for indicating excessive FPD prolongation prior to the induction of EAD/TAs. This is an important finding, since it is a major mechanism of TdP [23]. Interestingly, the threshold of FPD (500 ms) is close to the QT value of patients who developed TdP after treatment with dofetilide or sotalol: the mean QT values in lead II ECG analysis are 489 vs. 413 ms in patients with and without TdP, respectively [24]. This result indicates that the threshold of FPD/FPDcF prolongation is a potential marker for predicting the onset of human TdP, as shown in a previous report [25]. Further studies with more reference compounds and other types of hiPSC-CMs will be required to validate this finding.

The reasons why human-relevant characteristics were obtained in this study probably come from the high density cardiomyocyte sheets used for recording FP, rather than the innate electrophysiological properties of individual cells which are known to be variable. It is well-known that hiPSC-CMs are a mixture of subtypes of cardiomyocytes (nodal, atrial and ventricular) in which the expression of cardiac ion channels is also diverse. Therefore, patch clamp analyses of single cardiomyocytes show a large variation in drug effect, making it difficult to accurately interpret the risk of arrhythmogenicity of test compounds. On the other hand, FP recording using the MEA technique requires hiPSC-CM sheets in which each cell is electrically connected to its neighbors via tight-junctions. FP recording electrodes detect electrical changes from a large cell population and the FP signals represent the electrical average of these cells [18]. Therefore, FP recordings using MEA technology may be comparable to ECG measurements. It is noted that different results may be obtained using other types of hiPSC-CMs because the proportion of each cardiac cell-subtype or each cardiac ion-channels on the individual cardiomyocytes will be different among hiPSC-CMs from different sources. Further analyses are necessary to examine whether the human-relevant phenotypes are widely observed in hiPSC-CMs from other sources.

In conclusion, our results showing repolarization delay, reverse use-dependency, categorical analyses and the incidence of EAD/TAs demonstrate that hiPSC-CMs exhibit clinically relevant electrophysiological properties and thus may provide more reliable results than animal models for assessing the human arrhythmogenic potential of drug candidates in human.

## Supporting Information

S1 Table Baseline data among facilities. (DOCX)

S2 Table Effects of test drugs on field potential duration and incidence of early afterdepolarization or triggered activity. (DOCX)

S3 Table A corrected formula yielded by the linear regression model at each test drug. (DOCX)

S4 Table Summary of categorical analysis of delta FPD values. (DOCX)

S5 Table Summary of categorical analysis of delta FPDcF values. (DOCX)
