# Supplementary material for: Electrophysiological Characteristics of Human iPSC-Derived Cardiomyocytes for the Assessment of Drug-Induced Proarrhythmic Potential
Source: PLoS One. 2016 Dec 6;11(12):e0167348. doi: 10.1371/journal.pone.0167348 (PMC5140066; doi:10.1371/journal.pone.0167348)
Supplement: S3 Table — (DOCX) [file pone.0167348.s003.docx]

S3 Table. A corrected formula yielded by the linear regression model at each test drug.
