# Supplementary material for: Electrophysiological Characteristics of Human iPSC-Derived Cardiomyocytes for the Assessment of Drug-Induced Proarrhythmic Potential
Source: PLoS One. 2016 Dec 6;11(12):e0167348. doi: 10.1371/journal.pone.0167348 (PMC5140066; doi:10.1371/journal.pone.0167348)
Supplement: S4 Table — (DOCX) [file pone.0167348.s004.docx]

S4 Table. Summary of categorical analysis of delta FPD values.
